# CELL-E: A Text-To-Image Transformer for Protein Localization Prediction

**DOI:** 10.21203/rs.3.rs-2963881/v1

**Published:** 2023-06-02

**Authors:** Emaad Khwaja, Yun S. Song, Bo Huang

**Affiliations:** 1 UC Berkeley - UCSF Joint Graduate Program in Bioengineering, CA, USA.; 2 Computer Science Division, UC Berkeley, Berkeley, 94720, CA, USA.; 3 Department of Statistics, UC Berkeley, Berkeley, 94720, CA, USA.; 4 Chan Zuckerberg Biohub - San Francisco, San Francisco, 94158, CA, USA.; 5 Department of Pharmaceutical Chemistry, UCSF, San Francisco, 94143, CA, USA.; 6 Department of Biochemistry and Biophysics, UCSF, San Francisco, 94143, CA, USA.

**Keywords:** text-to-image synthesis, transformers, single-cell imaging, generative models

## Abstract

Accurately predicting cellular activities of proteins based on their primary amino acid sequences would greatly improve our understanding of the proteome. In this paper, we present CELL-E, a text-to-image transformer model that generates 2D probability density images describing the spatial distribution of proteins within cells. Given an amino acid sequence and a reference image for cell or nucleus morphology, CELL-E predicts a more refined representation of protein localization, as opposed to previous *in silico* methods that rely on pre-defined, discrete class annotations of protein localization to subcellular compartments.

## 1 Introduction

In recent years, advancements in sequencing technologies have allowed for the comprehensive cataloging of proteins and their amino acid sequences across a wide range of organisms [1]. Despite this progress, the exact functions and cellular dynamics of many proteins remain unclear. In order to gain a deeper understanding of these proteins, researchers have sought ways to predict their properties, including structure, interactions, subcellular localization, and trafficking patterns, from their amino acid sequences. This type of computational analysis has the potential to shed light on the “dark matters” of the proteome and enable large-scale screening before expensive experimental validation. These tools have numerous applications in biomedical research, such as drug design and therapeutic target discovery [2].

In this study, our focus is on predicting subcellular localization of proteins from their amino acid sequences, which serves as the spatial context for their cellular functions. The localization of a protein to a specific subcellular compartment can be driven by either active transport or passive diffusion in conjunction with specific protein-protein interactions, often involving localization “signals” in the amino acid sequence [3–5]. In many cases, however, the exact mechanisms for sequence recognition and trafficking are not yet fully understood [6]. For example, there is ongoing debate about the mechanism behind the import of proteins via the nuclear localization sequence (NLS) [7]. Given these challenges, machine learning utilizing existing knowledge of protein localization has become a particularly useful tool.

Although computational prediction of protein subcellular localization from primary amino acid sequences is an active area of research, most works train the model with class annotation of subcellular compartments (e.g., nucleus, plasma membrane, endoplasmic reticulum, etc.) [8] which are available from databases such as UniProt [9]. This approach has two major limitations. First, many proteins are present in different and variable amounts across multiple subcellular compartments. Second, protein localization could be highly heterogeneous and dynamic depending on the cell type and cell state (including cell cycle state). Neither of these two aspects have been captured by existing discrete class annotations. Consequently, machine-learning-based protein localization prediction still has limited applications. Furthermore, to assist mechanistic discoveries, it is highly desirable for the machine learning models to be explainable.

**Fig. 1:**
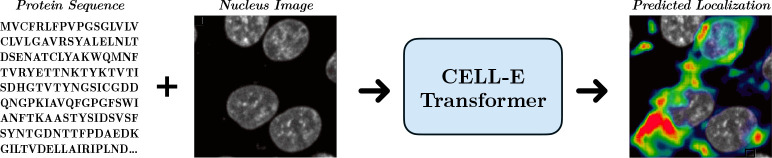
Given an amino acid sequence and a reference nucleus image, CELL-E makes a prediction of protein localization with respect to the nucleus as a 2D probability density function, shown as heatmap, with color indicating relative confidence for each pixel.

To investigate the relationship between sequence and subcellular localization, we present CELL-E, a text-to-image transformer model which predicts the probability of protein localization on a per-pixel level from a given amino acid sequence and a conditional reference image for the cell or nucleus morphology and location (Fig. 1). It relies on transfer learning via amino acid embeddings from a pre-trained protein language model and two quantized image encoders trained from a live-cell imaging dataset. By generating a two-dimensional probability density function (2D PDF) atop the reference image, CELL-E naturally accounts for multi-compartment localization and the cell type/state information implicitly encoded by the cell morphology. We demonstrate the capability of CELL-E to predict localization of proteins, identify changes in localization due to mutations, and uncover sequence features correlated with the specification of subcellular protein localization.

## 2 Results

### 2.1 The CELL-E Model

**Fig. 2:**
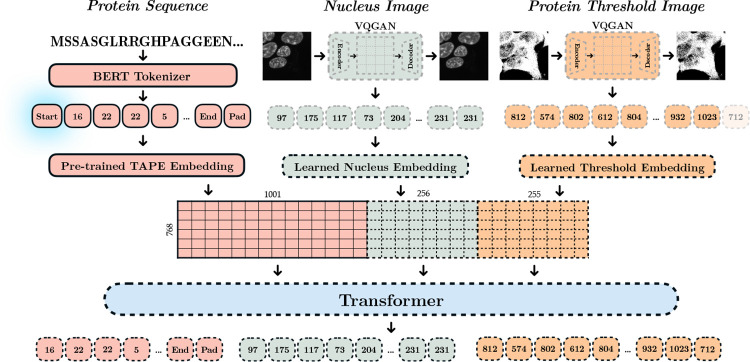
Graphical depiction of CELL-E. Solid lines correspond to pre-trained components. Gray dashed lines are learned in Phase 1 and 2 (Reference Image and Protein Threshold VQGANs). Black dashed lines correspond to components learned in Phase 3. A start token is prepended to the sequence and the final protein image token is removed. The amino acid sequence embedding from the model is preserved, and embedding spaces for the image tokens are cast in the same depth and concatenated with the amino acid sequence embedding. The transformer is tasked with reproducing the original sequence of tokens (e.g., the input sequence with start token shifted to the right one position).

CELL-E (Fig. 2) is inspired by the text-to-natural-image generation model of DALL-E [10] (See Section S.2.1 for a review of relevant work). Similar to DALL-E, our model autoregressively learns text and image tokens as a single stream of data. On the other hand, While the goal of general text-to-image models is to produce images with high perceptual strength, they do not necessarily aim for quantitative accuracy [10–12]. Therefore, CELL-E was designed with the following considerations:

#### 1. Transfer learning.

Training CELL-E requires a library of cellular images and corresponding morphological reference images for a large number of proteins. For this purpose, we utilized the recently established OpenCell library[13], which contains a library of 1,311 CRISPR-edited HEK293T human cell lines, each having one target protein fluorescently tagged and imaged by confocal microscopy with accompanying DNA staining as the reference for nuclei morphology. The high image quality and consistency makes OpenCell a good choice as the training and validation dataset (See Section S.3.1 for more information). Still, data availability in this domain remains a large obstacle. For example, DALL-E was trained on 250 million text-images pairs [10], orders of magnitude larger than the OpenCell dataset. We utilize transfer learning by incorporating frozen embeddings from a pre-trained protein language model as the input representation of the amino acid text sequence. This approach reduces the number of learned paramaters, thereby alleviating the burden for CELL-E to also learn the amino acid sequence space. This allows training to be concentrated on the relationship between sequence and image tokens. We evaluated multiple protein language models (see Supplementary Information and Table S1) and eventually chose the BERT-based model from Rao et. al. [14], which we refer to as the TAPE model, for subsequent work.

#### 2. Morphological reference.

In our initial efforts, we found that a transformer using just the amino acid tokens and protein image tokens is capable of generating cell-like images from the amino acid sequence alone (Fig. S3). However, quantifying protein localization information in the generated images is challenging. Furthermore, an estimation of a single snapshot of protein localization is not necessarily a quantifiable indication of global behavior. Therefore, in addition to amino acid tokens and protein image tokens, we add a 3rd embedding space to include tokens representing the overall cell morphology from a reference image. The reference image provides the model with information regarding the localization of subcellular structures and compartments. Moreover, cell morphology implicitly provides the cell type and cell state context for CELL-E predictions.

#### 3. Image model.

Instead of the Vector Quantized Varational Autoencoder (VQVAE) previously used to analyze OpenCell imaging data [15], we chose to use Vector Quantized Generative Adversarial Network (VQGAN) [16] which produces images with comparatively higher spatial frequency. To simplify the task of the protein image VQGAN, we let it predict per-pixel binary representations of protein localization (i.e., a thresholded image). This allows us to use the marginal probabilities predicted for each image token from CELL-E to create a weighted sum on the image tokens. This latent space linear combination is then used to generate a continuous 2D probability density function of protein localization, which resembles a gray-scale image (Fig. 7). We note that the same model can also be trained to output gray-scale images directly (See Supplementary Notes S.2.2 and Fig. S4).

### 2.2 Performance Evaluation

**Fig. 3:**
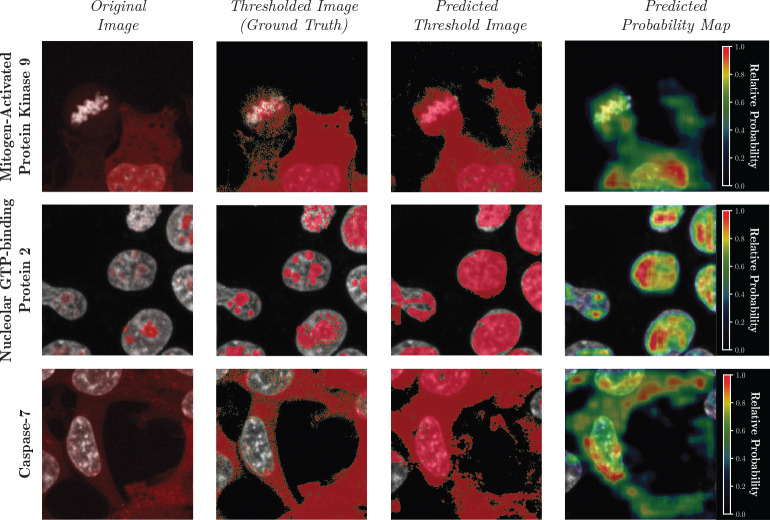
Prediction results of several types of proteins from the validation set, unseen to the model during training. The nucleus channel is depicted in grayscale, and the protein channel is shown as an overlay in red (Fig. S1 for clarification). The thresholded image (Column 2) is designated “Ground Truth” because those are the types of images exposed to the model during training. The predicted probability map is obtained from a weighted sum of potential image patches and normalized to 1.

Fig. 3 and Fig. S2 show the CELL-E predictions for several proteins in the validation dataset. High similarities can be seen between the predictions and the ground truth. Even though the reference images only depict the nuclei, which is a limitation of the OpenCell training data, CELL-E can reasonably paint the shape of the cell for cytoplasmic proteins. Interestingly, the case of Mitogen-Activated Protein Kinase 9 (MAPK9) contains a cell in metaphase (top row of Fig. 3). CELL-E correctly predicts the round shape of its distribution around the mitotic chromosomes instead of the more expanded distribution for the adjacent interphase cell. This result suggests that CELL-E can indeed capture cell state information from the morphological reference images.

We used several metrics to evaluate the reconstruction performance of CELL-E, summarized in Table S2. Among the metrics, nucleus proportion accuracy measures how close the estimated proportion of pixel intensity within the nucleus is to the ground truth thresholded image. We believe this is the most relevant metric as it is not obscured by small spatial variations and nucleus boundaries can be obtained from the reference images. Description of other metrics and more information on the evaluation procedure can be found in Section S.3.7. Using these metrics, we performed ablations studies to optimize our model architecture and choice of protein language embedding (see Section S.2.3, Fig. S5 and Table S1).

While CELL-E is not specifically trained as a discrete localization classifier, we also performed naive comparison between CELL-E model and 1D protein localization classifiers MuLoc [17] and Subcons [18] specifically trained with annotated protein localizations. We focused on nuclear classification using a simple classification criteria on CELL-E output (see Section S.3.7), and the results are summarized in Table 1. We observed a relatively high degree of accuracy from this method compared to the task-specific models. CELL-E was a close second for validation set proteins despite not seeing localization annotations during training.

### 2.3 Analysis of NLS using CELL-E

**Fig. 4:**
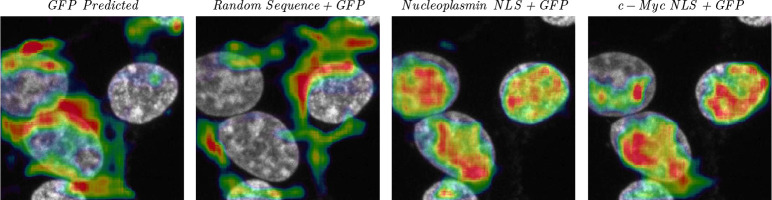
Predicted localization of GFP and modified-GFP sequences.

As a first test to show that CELL-E can recognize specific, functional sequence features, we let it predict the images for Green Fluorescent Protein (GFP), which is non-native to human and does not contain known localization signals, as well as GFP appended with two commonly used NLS’s KRPAATKKAGQAKKKK from nucleoplasmin [19] and PAAKRVKLD from N-Myc [20]) that drive nuclear localization of a protein. We also appended a randomly generated sequence as a control. A randomly chosen nuclear image from the OpenCell dataset was used as the morphological reference. CELL-E does not localization of GFP (or random sequence + GFP) to a specific subcellular compartment with high confidence, whereas the two NLS-GFP fusions are clearly predicted to be localized within the nucleus (Fig. 4). Therefore, CELL-E has the potential to perform computational insertion screenings for the functional sufficiency of putative localization sequence features.

**Fig. 5:**
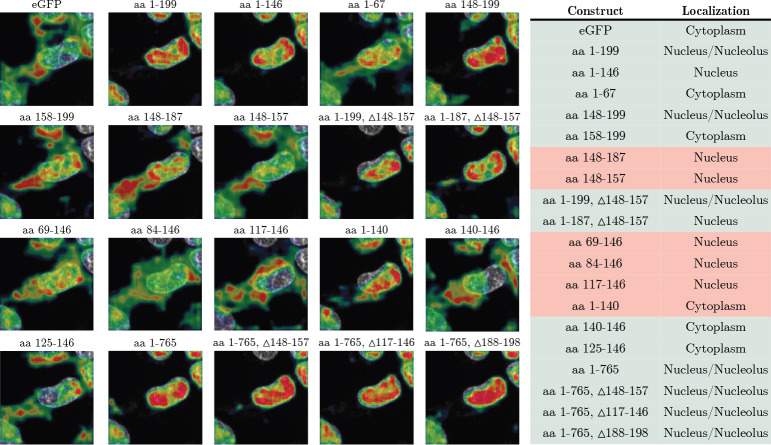
CELL-E’s predicted localization (images) of eGFP fusions from [22] and corresponding localization annotations (table) from the original paper. In the table on the right hand side, green indicates agreement between CELL-E and experimental results, while red indicates disagreement. *aa 1–199* contains the entire N-terminus region. *aa 1–146* only contains Motifs I and V. *aa 1–67* only contains Motif-I. *aa 148–199* contains Motif II, III, IV and V.

Next, we examined whether CELL-E can identify NLS in a protein by computationally performing truncation/deletion studies. For this purpose, we chose DNA Topoisomerase I (TOP1), whose N-terminal intrinsically disordered region (amino acid (aa) 1–199) is essential for its nuclear localization [21]. An experimental study generated a series of deletion mutants for this region and imaged the subcellular localization in HeLa cells when fused to eGFP [22]. To computationally reproduce this study, we fed the exact sequences of the deletion mutants to CELL-E. As shown in Fig. 5, the predictions were largely consistent with the experimental data, recapturing the inability for *aa 1–67* to drive nuclear localization despite containing a putative NLS, as well as the sufficiency of *aa 148–199* as an NLS.

**Fig. 6:**
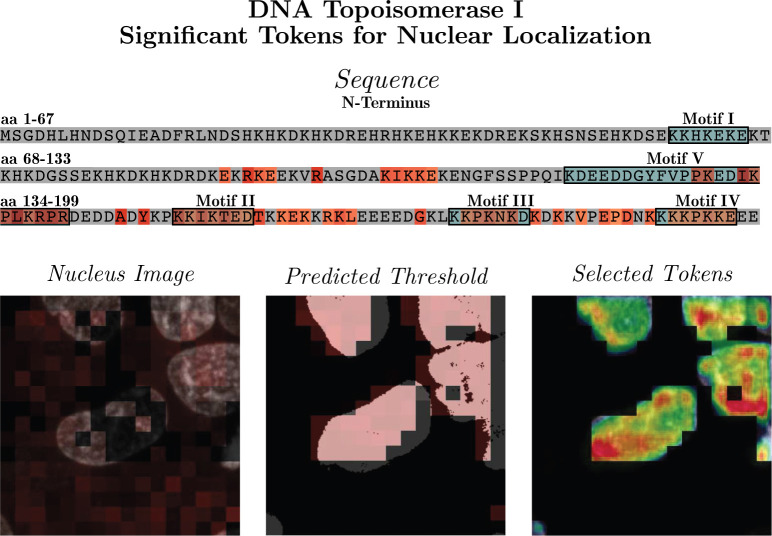
Attention weights for significant tokens when patches containing a large percentage of protein are selected (bottom-right figure). Previous computationally identified putative NLSs are boxed in black (top figure). These are *aa 59–65* (Motif I, KKHKEKE), *aa 150–156* (Motif II, KKIKTED), *aa 174–180* (Motif III, KKPKNKD), and aa 192–198 (Motif IV, KKKPKKE). Additionally, the new NLS identified in Mo et. al.[22], Motif V (*aa 117–146* ), is highlighted.

Lastly, we demonstrate a more direct approach than computational insertion or deletion studies to identify putative sequence features responsible for protein localization. Specifically, we split the generated image patches into two groups, one with the target protein being present and the other being absent based on the average pixel intensity within the 16 × 16 image patch. Then, we calculated the difference of attention weights for each amino acid token to contribute to the two groups. Fig. 6 highlights the amino acids with higher weights for the “present” group. The highlighted amino acids include the three putative NLSs (Motifs II, III, and IV) in the experimentally verified *aa 148–199* range, as well as part of the new *aa 117–146* NLS identified in [22]. On the other hand, the putative NLS (Motif I) in the experimentally invalidated *aa 1–69* range are not activated. The attention map also suggest that *aa 89–107* (KIKKE) could be another NLS in this protein. We must point out that the calculation of attention map was simply based on a protein being “present” or “not present” in image patches and did not specify “nuclear localization” at all. Therefore, it should be capable of serving as a general approach to discover putative sequence features driving protein localization to a variety of subcellular compartments.

## 3 Discussion

CELL-E’s performance seems to be currently limited by the scope of the OpenCell dataset, which only accounts for a handful of proteins within a single cell type and imaging modality. As the OpenCell project is an active development, we expect stronger performance as more data become available. The availability of brightfield (e.g., phase-contrast) images as the morphological reference will also likely improve the prediction of cytoplasmic protein localization compared to using nuclei images. Furthermore, the utility of the model comes in terms of linking embedding spaces of dependent data. One could imagine follow up experiments where rather than images being the prediction, other signatures such as protein mass spec could be predicted. Additionally, other sources of information, such as structural embeddings could be incorporated to bolster CELL-E’s capabilities.

## 4 Methods

We use a multi-phase training approach similar to DALL-E, but our model also uses pre-trained language-model input embeddings for the amino acid text sequences via TAPE:

- **Phase 1** A Vector Quantized-Generative Adversarial Network (VQGAN) [16] is trained to represent a single channel 256 × 256nucleus image as a grid comprised of 16 × 16 image tokens (Fig. S7), each of which could be one of 512 tokens.
- **Phase 2** A similar VQGAN is trained on images corresponding to binarized versions of protein images. These tokens represent the spatial distribution of the protein (Fig. S9).
- **Phase 3** The VQGAN image tokens are concatenated to 1000 amino acid tokens for the autoregressive transformer which models a joint distribution over the amino acids, nucleus image, and protein threshold image tokens.

### 4.1 Model Specifics

The optimization problem is modelled as maximizing the evidence lower bound (ELBO) [23, 24] on a joint likelihood distribution over protein threshold images u, nucleus images x, amino acids y, and tokens z for the protein threshold image:

#### Theorem 1.

$$p_{\theta ,\psi }(u,x,y,z)=p_{\theta }(u|x,y,z)p_{\psi }(x,y,z)$$

This is bounded by:

#### Theorem 2.

$$\ln\;p_{\theta ,\psi }(u,x,y)\underset{z\sim q_{\phi }(z|u)}{\geq E}\left[\ln\;p_{\theta }(u|x,y,z)\right]-KL\left(q_{\phi }(x,y,z|u),p_{\psi }(x,y,z)\right)$$

where $q_{\phi }$ is the distribution 16 × 16 image tokens from the VQGAN corresponding to the threshold protein image $u,\;p_{\theta }$ is the distribution over protein threshold generated by the VQGAN given the image tokens, and $p_{\psi }$ indicates the joint distribution over the amino acid, nucleus, and protein threshold tokens within the transformer.

### 4.2 Nucleus Image Encoder

Training both image VQGANs maximizes ELBO with respect to ϕ and θ. The VQGAN improves upon existing quantized autoencoders by introducing a learned discriminator borrowed from GAN architectures [16]. The Nucleus Image Encoder is a VQGAN which represents 256 × 256 nucleus reference images as 256 16 × 16 image patches. The VQGAN codebook size was set to n=512 image patches. Further details can be found in Section S.3.4.

### 4.3 Protein Threshold Image Encoder

The protein threshold image encoder learns a dimension reduced representation of a discrete binary PDF of per-pixel protein location, represented as an image image. We adopt a VQGAN architecture identical to the Nucleus VQGAN. The VQGAN serves to approximate the total set of binarized image patches. While in theory a discrete lookup of each pixel arrangement is possible, this would require ~ 1.16 × 10^77^ entries, which is computationally infeasible. Furthermore, some distributions of pixels might be so improbable that having a discrete entry would be a waste of space.

Protein images are binarized with respect to a mean-threshold, via:

$${\overset{‾}{u}}_{i,j}=\left\{\begin{array}{ll} 1, & u_{i,j}\geq \mu ,\\ 0, & u_{i,j}<\mu , \end{array}\right.$$

∀ pixels u∈ image U of size i×j, where μ is the mean pixel intensity in the image (Fig. S8).

The 16 × 16 image patches learned within the VQGAN codebook therefore correspond to local protein distributions. In Section 4.6, we detail how a weighted sum over these binarized image patches is used to determine a final probability density map. Hyperparameters and other training details can be found in Section S.3.5.

### 4.4 Amino Acid Embedding

For language transformers, it is necessary to learn both input embedding representations of a text vector as well as attention weights between embeddings [25]. In practice, this creates a need for very large datasets [26]. The OpenCell dataset contains 1,311 proteins, while the human body is estimated to contain upwards of 80,000 unique proteins [27]. It is unlikely that such a small slice could account for the large degrees of variability found in nature.

In order to overcome this obstacle, we opted for a transfer learning strategy, where fixed amino acid embeddings from a pre-trained language model exposed to a much larger dataset were utilized. We found the strongest performance came from TAPE embeddings [14]. Utilizing pre-trained embeddings had the two-fold benefit of giving our model a larger degree of protein sequence context, as well as reducing the number of trained model parameters, which allowed us to scale the depth of our network.

We tried training using random initialization for amino acid embeddings (See Section S.2.3), however, we noted overfitting on the validation set image reconstruction and high loss on validation sequences. We also experimented with other types of protein embeddings, including UniRep [28] and ESM1-b [29].

### 4.5 CELL-E Transformer

The transformer $\left(p_{\phi }\right)$ utilizes an input comprised of amino acid tokens, a 256 × 256 nucleus image crop, and the 256 × 256 corresponding protein image threshold crop. In this phase, ϕ and θ are fixed, and a prior over all tokens is learned by maximizing ELBO with respect to ϕ. It is a decoder-only model [30].

The model is trained on a concatenated sequence of text tokens, nucleus image tokens, and protein threshold image tokens, in order. Within the CELL-E transformer, image token embeddings were cast into the same dimensionality as the language model embedding to in order to maintain the larger protein context information, however the embeddings corresponding to the image tokens within this dimension are learned (See Fig. 2).

### 4.6 Probability Density Maps

When generating images, the model is provided with the amino acid sequence and nucleus image. The transformer autoregressively predicts the protein-threshold image. In order to select a token, the model outputs logits which contain probability values corresponding to the codebook identity of the next token. The image patch $v_{i}$ is selected by filtering for the top 25% of tokens and applying top-k sampling with gumbel noise [31].

Ordinarily, the final image is generated by converting the predicted codebook indices of the protein threshold image to the VQGANs decoder. However, to generate the probability density map $\overset{‾}{v}$, we include the full range of probability values corresponding to image patches, $p\left(v_{i}\right)$, obtained from the output logits. The values are clipped between 0 and 1 and multiplied by the embedding weights within the VQGAN’s decoder, $w_{i}$:

#### Theorem 3.


$$\overset{‾}{v}=w\cdot p(v)=\sum_{i=1}^{n}w_{i}\;p\left(v_{i}\right)$$

**Fig. 7:**
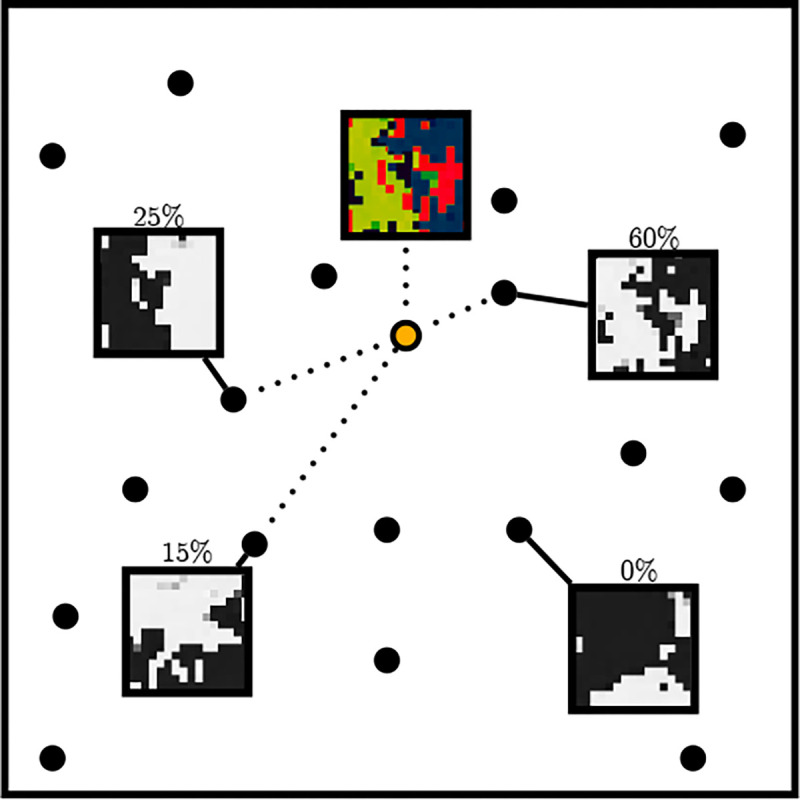
Simplified example of probability map calculation. Each circle corresponds to an image token within the quantized VQGAN embedding space. Each PDF patch (yellow) is obtained as a weighted sum over all protein threshold image VQGAN codebook vectors.

This output is normalized and displayed as a heatmap (Fig. 7).

## Supplementary Material

Supplement 1

## Figures and Tables

**Table 1:** Nuclear Localization Prediction Accuracy

	Train	Validation
		
VQGAN	0.99 ± 0.08	0.99 ± 0.09

CELL-E	**0.89 ± 0.31**	0.72 ± 0.45
MuLoc	0.71 ± 0.45	**0.79 ± 0.41**
Subcons	0.43 ± 0.49	0.69 ± 0.46

VQGAN indicates the accuracy evaluated on the ground truth threshold image passed through the VQGAN image encoder. As CELL-E selects tokens from this VQGAN to produce its outputs, these values represent the best possible performance for our model.
