# Supplementary material for: CELL-E: A Text-To-Image Transformer for Protein Localization Prediction
Source: Res Sq. 2023 Jun 2:rs.3.rs-2963881. Preprint. [Version 1] doi: 10.21203/rs.3.rs-2963881/v1 (PMC10312902; doi:10.21203/rs.3.rs-2963881/v1)
Supplement: Supplement 1 [file NIHPPRS2963881V1-supplement-1.pdf]

Appendix S.1 Supplementary Figures

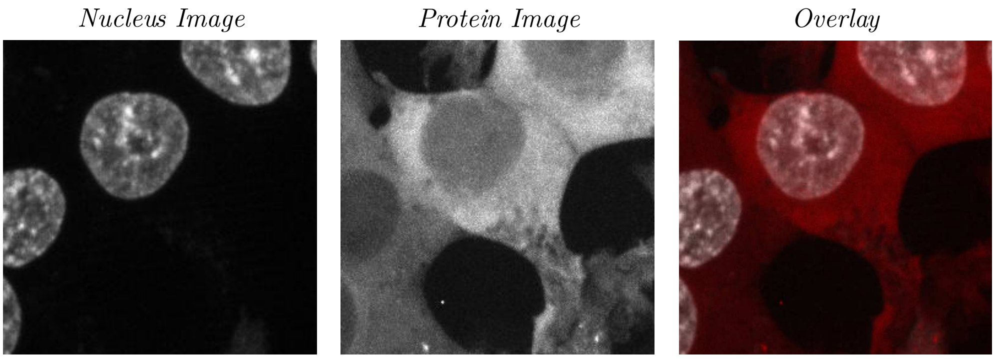

Fig. S1 Nucleus Image (left), Protein Image (middle), and Overlay (Right). The alpha value for the protein channel in the right column is set to .7. Overlay is used as the “Original Image” in Fig. 3 and Fig. S2.

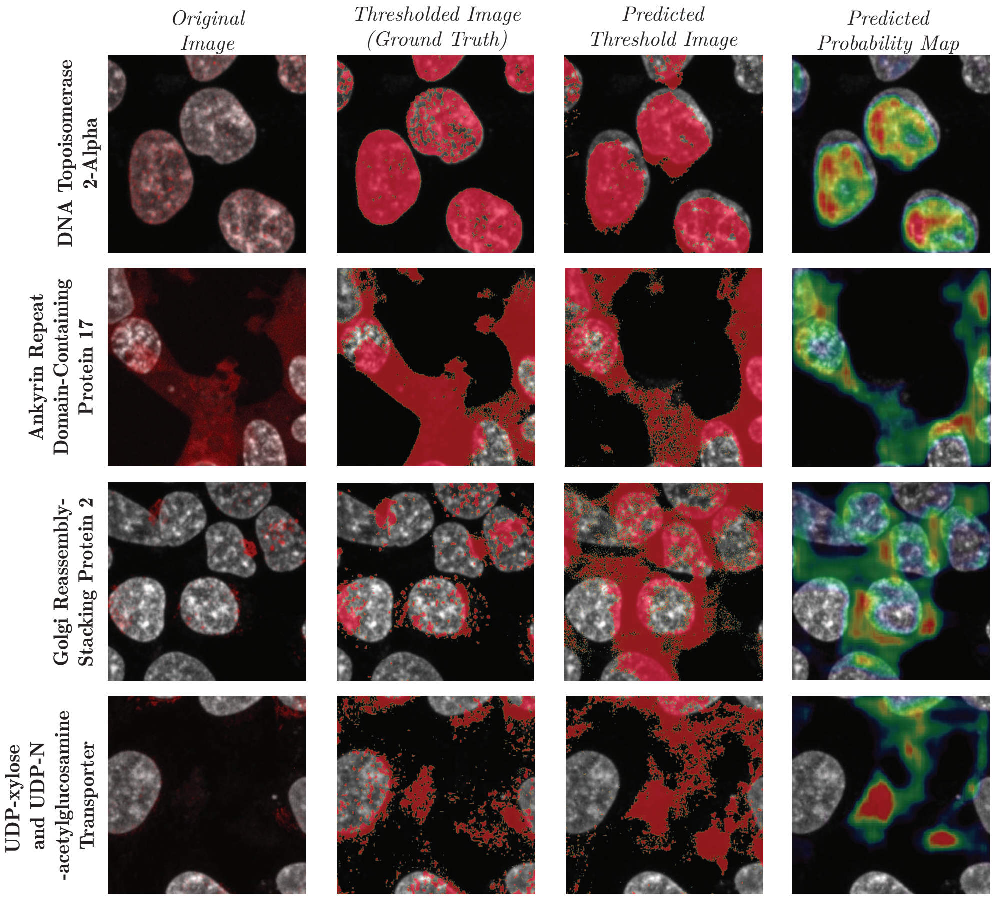

Fig. S2 More prediction results from the validation set. We observe a high degree of spatial awareness from the model, notably in UDP-xylose-acetylglucosamine Transporter, which accurately predicts signal between cell nuclei with high confidence.

Appendix S.2 Supplementary Notes

S.2.1 Related work

Natural language processing (NLP) has found applications in amino acid sequence encoding, due to the long contextual dependencies of amino acids in a protein's folded three-dimensional structure [33]. Self-supervised models from the NLP field have demonstrated excellent performance in predicting protein properties from amino acid sequence inputs [29, 34–36]. These models are trained on millions of amino acid sequences from databases such as BFD [37], UniRef [38], Pfam [39], and Protein Data Bank [40]. The language models have proven effective in downstream tasks like structure prediction, evolutionary analysis, and protein engineering [14], with LSTM and attention-based models achieving particularly impressive results [25]. UniRep is an

LSTM model that predicts the next amino acid in a variable length sequence [41], while BERT uses bidirectional masked language modeling to predict the identity of masked tokens throughout the sequence [42]. Facebook's Evolutionary Scale Model (ESM) is a state-of-the-art masked-language model model, pre-trained with 250 million amino acid sequences and over 700 million parameters [29].

While traditional supervised approaches, such as stochastic modeling, have been limited by feature representation or computation time, deep learning has proven to be a powerful tool in predicting localization [43]. With the ability to optimize millions of parameters, deep neural networks have shown the ability to represent complex patterns in a manner that traditional manual feature extraction cannot [44, 45].The success of language models in protein prediction tasks suggests that patterns dictating these structures are buried within residue sequences [14, 28].

Protein localization is typically framed as a class prediction task. 1D localization predictors take the primary sequence as input and produce a fixed-length vector, with each entry corresponding to a subcellular location and the values being probability values. However, these methods have limitations [46, 47]. Discrete classifications for contiguous regions of the cell, such as the nuclear membrane, can be ambiguous and may have flawed annotations in established datasets. [48]. Additionally, these methods do not account for the influence of local cellular geometries [49–52] and cell states [53, 54] on transport dynamics. For example, one would expect significantly high amounts of transcription factors for DNA replication in the S-phase of the cell cycle, but not during cell separation in mitosis [55]).

S.2.2 Text-To-Image Generation

Ramesh et. al. [10] demonstrated true zero-shot text-to-image generation with their model, DALL-E. Unlike previous models, DALL-E utilized an autoregressive framework, which was trained on a joint distribution of text and image tokens, enabling it to make novel image predictions with high fidelity. In contrast, earlier models based on variational autoencoders (VAE) [23] or Generative Adversarial Networks (GAN) [56] performed poorly when generating images outside of the training data, resulting in distorted images and artifacts [57–59].

While our method does similarly allow for truly zero-shot protein image prediction (Fig. S3), our goal for image generation extends beyond visual fidelity and includes a degree of spatial accuracy. This is crucial for capturing the dynamic process of protein abundance in cells, which fluctuates with respect to cell state and environmental factors. To overcome this challenge, the model is tasked with predicting per-pixel binary probability representations of protein localization, which can then be linearly combined to generate a continuous 2D probability density function of protein localization.

The following images (Fig. S3, Fig. S4) are from text-to-image models architecturally similar to CELL-E, but replace the Protein Threshold VQGAN with a similarly trained Protein Image VQGAN.

Fig. S4 shows model outputs model similar to Fig. S3 above, but does include image synthesis conditioned on a nucleus image input (via Nucleus Image VQGAN). The predicted outputs are perceptually more similar to the ground truth protein image,

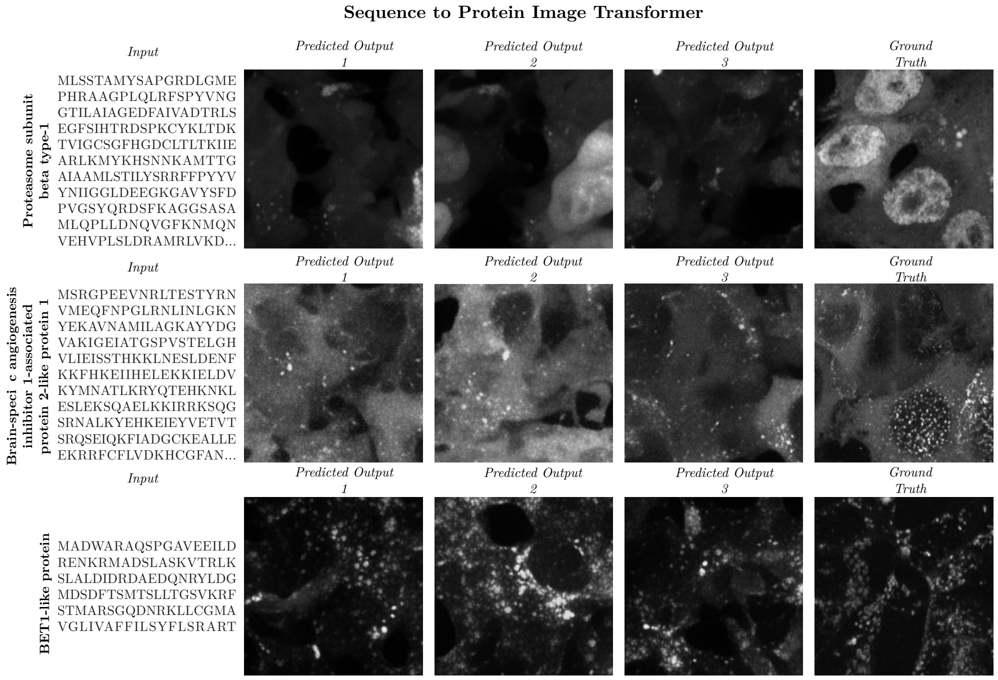

Fig. S3 DALL-E-like model with only amino acid sequence as the input. The sequence (left column) is used as input. The middle 3 columns show separate predicted images from random initialization. The true protein image is shown in the right column.

but the questions of accuracy and scientific utility mentioned previously continue to be a factor within this paradigm.

The sequence-to-image transformer is similar to CELL-E and does not take a nucleus image as input. This produces perceptually similar images (Fig. S3). Within the training data, the corresponding protein image is merely a snapshot in time and could be markedly different if taken at another time point. For this reason, we do not believe a image prediction without confidence provides scientific utility.

S.2.3 Ablation Study

Model Architecture In order to gauge the importance of each component of the model, we performed ablation by training several versions of the model with the same initialization. We specifically chose to look at performance in nuclear proportion accuracy over the validation set (Fig. S5).

On the top row, performance from the VQGAN is used as a reference of possible top performance, just as in Table S2. The second row depicts our main CELL-E (with depth = 32 and a fixed language embedding) used for this study. We found the performance in both cross-entropy and nuclear proportion accuracy increased with model depth when compared to similar models of smaller depth (third, fourth, and fifth rows).

Sequence Embedding Model	Model Depth	Embedding Dimension	Fixed Sequence Embedding?	Epochs Trained	Nucleus Proportion Accuracy	Predicted Threshold Pixel Accuracy	Predicted 2D PDF Pixel Accuracy	SSIM	IS	FID	Nucleus Localization Prediction Accuracy
**Train**											
N/A (VQGAN)				371	.9927 ± .0084	.8749 ± .0817		.5523 ± .2528	4.174 ± .1716	14.818	.9933 ± .0810
TAPE	32	768	✓	130	.9396 ± .0535	.7678 ± .0647	.6758 ± .1047	.3154 ± .2100	2.7684 ± .0681	107.4147	.8946 ± .3071
TAPE	26	768	✓	136	.9475 ± .0465	.7551 ± .0726	**.6849 ± .1051**	.3146 ± .2195	2.9021 ± .0067	81.0061	.9059 ± .2920
TAPE	26	768	✗	49	.9260 ± .0656	.7568 ± .0664	.6131 ± .0928	.2266 ± .1485	2.3797 ± .0742	140.339	.8730 ± .3330
TAPE	20	768	✓	63	.9433 ± .0501	.7647 ± .0719	.6815 ± .1081	.3138 ± .2234	2.8539 ± .0474	98.5444	.9004 ± .2994
TAPE	15	768	✓	84	.9456 ± .0469	.7485 ± .0704	.6730 ± .1041	.2955 ± .2122	2.687 ± .0742	112.4738	**.9095 ± .2869**
TAPE (No Nuc.)	32	768	✓	91	.9115 ± .0762	.7609 ± .0762	.5743 ± .0887	.2092 ± .1609	2.2793 ± .3975	137.8209	.8516 ± .3554
ESM1b	20	1280	✓	54	.9403 ± .0523	.7610 ± .0664	.6633 ± .1027	.2855 ± .2057	2.6059 ± .1153	111.8584	.8902 ± .3127
UniRep	15	1900	✓	8	.6591 ± .1547	.7420 ± .0632	.6011 ± .0803	.2108 ± .1410	2.1317 ± .0432	193.1455	.8651 ± .3417
AA Descriptors	58	66	✓	60	.9439 ± .0528	.7674 ± .0690	.6815 ± .1083	.3220 ± .2261	2.8121 ± .0529	94.2488	.9044 ± .2941
AA Descriptors	32	66	✓	66	.9472 ± .0499	.7622 ± .0792	.6825 ± .1114	**.3293 ± .2420**	**3.144 ± .1315**	**65.5666**	.9082 ± .2888
One-Hot	59	25	✓	100	.9469 ± .0487	.7524 ± .0681	.6791 ± .1077	.3141 ± .2212	2.7269 ± .0917	102.8544	.9068 ± .2907
One-Hot	32	25	✓	70	**.9478 ± .0476**	.7475 ± .0705	.6769 ± .1073	0.3041 ± .2287	2.7469 ± .0905	90.0424	.9057 ± .2922
Random Initialization	26	768	✗	71	.9348 ± .0567	**.7693 ± .0668**	.6606 ± .1093	.2943 ± .2144	2.7503 ± .0852	110.9421	,8885 ± .3147
No Sequence	32	768		51	.9235 ± .0678	.7578 ± .0749	.6113 ± .0930	.2196 ± .1517	2.2836 ± .0658	159.0451	.8628 ± .3440
**Validation**											
N/A (VQGAN)				371	0.9921 ± .0091	.8756 ± .0824		.5567 ± .2540	3.8718 ± .1662	25.9567	.9923 ± .0872
TAPE	32	768	✓	130	**.8078 ± .1837**	.7653 ± .0520	.6342 ± .0964	.2536 ± .1629	2.1300 ± .0704	155.7741	.7155 ± .4511
TAPE	26	768	✓	136	.7943 ± .1992	.7574 ± .0607	.6377 ± .1026	.2446 ± .1689	2.2708 ± .1303	136.294	.6979 ± .4594
TAPE	26	768	✗	49	.7300 ± .2229	.7505 ± .0648	.6092 ± .0918	.2148 ± .1436	2.2962 ± .1740	155.4038	.5905 ± .4917
TAPE	20	768	✓	63	.8024 ± .2071	**.7739 ± .0613**	**.6388 ± .1088**	.2547 ± .1821	2.2641 ± .1379	156.3681	.7017 ± .4575
TAPE	15	768	✓	84	.8044 ± .1927	.7422 ± .0554	.6267 ± .0914	.2226 ± .1546	2.0341 ± .0848	170.5773	**.7247 ± .4467**
TAPE (No Nuc.)	32	768	✓	91	.7742 ± .2239	.7733 ± .0660	.5688 ± .0739	.1956 ± .1311	**3.3204 ± .1737**	**45.6887**	.6288 ± .4833
ESM1b	20	1280	✓	54	.8044 ± .1851	.7465 ± .0602	.6235 ± .0916	.2207 ± .1604	2.1925 ± .0868	146.5215	.7224 ± .4478
UniRep	15	1900	✓	8	.7474 ± .2063	.7393 ± .0616	.5982 ± .0789	.2009 ± .1332	2.1287 ± .1231	206.3325	.6173 ± .4862
AA Descriptors	58	66	✓	60	.7854 ± .2234	.7642 ± .0602	.6340 ± .1020	.2487 ± .1736	2.2561 ± .1030	144.271	.7002 ± .4582
AA Descriptors	32	66	✓	66	.7688 ± .2435	.7676 ± .0761	.6350 ± .1163	**.2662 ± .2016**	2.8155 ± .1382	88.4847	.6817 ± .4658
One-Hot	59	25	✓	100	.7688 ± .1989	.7347 ± .0535	.6207 ± .0904	.2209 ± .1484	2.1214 ± .1182	159.35	.6457 ± .4783
One-Hot	32	25	✓	70	.7714 ± .2094	.7383 ± .0599	.6255 ± .1032	.2252 ± .1703	2.2802 ± .1170	129.3922	.6419 ± .4795
Random Initialization	26	768	✗	71	.7587 ± .2169	.7605 ± .0517	.6204 ± .0948	.2287 ± .1543	2.083 ± .1373	167.6092	.6250 ± .4841
No Sequence	32	768		51	.7140 ± .2359	.7528 ± .0733	.6054 ± .0889	.2032 ± .1387	2.2473 ± .1400	169.204	.5721 ± .4948

Table S1 Full Results Table

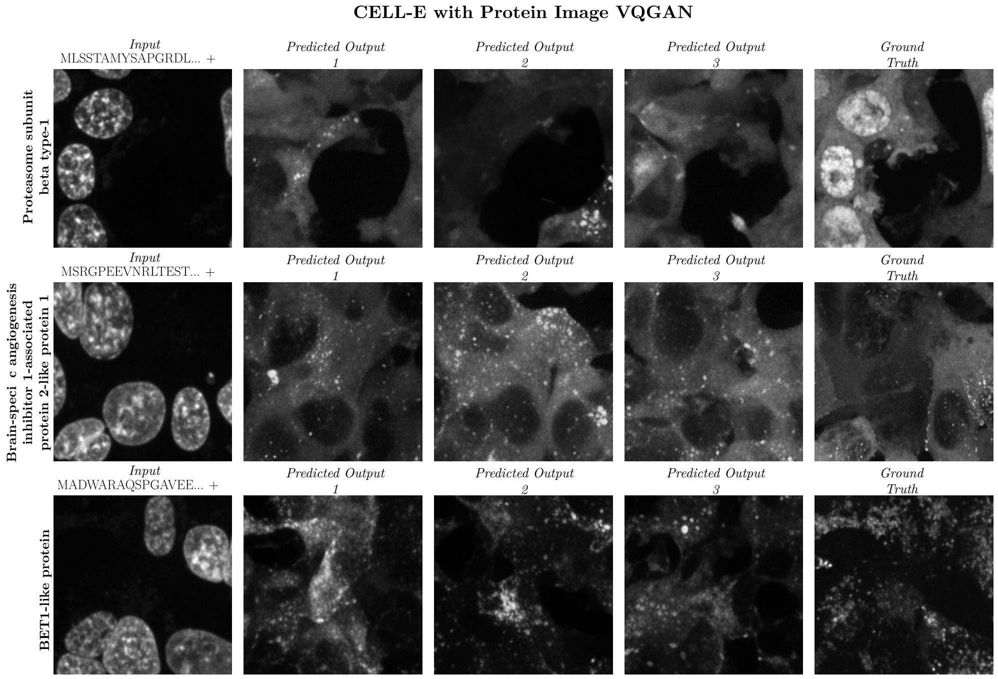

Fig. S4 CELL-E model with Protein Threshold VQGAN replaced with Protein Image VQGAN.

To understand the effect of using fixed language models, we trained 2 versions of CELL-E of same depth. The first (second row, light blue) had a fixed language embedding, while the second (seventh row, red) was free to change during training. We also introduced a model with a randomly initialized language embedding (sixth row, light green). While we note fairly high performance from the unfixed models on the training data, they performed quite poorly on the validation, indicating severe overfitting. This is a result of the comparatively small number of proteins represented within the OpenCell dataset when compared to the large pFam database used to train TAPE.

We also trained a versions of the model which did not use a nucleus input (second to last row, pink) like DALL-E, and a model that only used a nucleus input and no sequence (last row, purple), although a start token was still prepended.

Overall, we observe a distributional shift to the right, indicating more accurate predictions, as the depth of the transformer is scaled. Full results for both training and validation sets can be seen in Table S1. We also evaluated the performance of CELL-E using different protein embedding spaces. These were configured such that they were either at the same depth as the TAPE model, or the depth was scaled as deep as possible such that the GPU memory was saturated during training. All model were trained until convergence on the validation set.

Language Embedding Alongside language embeddings, we also used one-hot and amino acid chemical descriptors as embedding features. The amino acid chemical

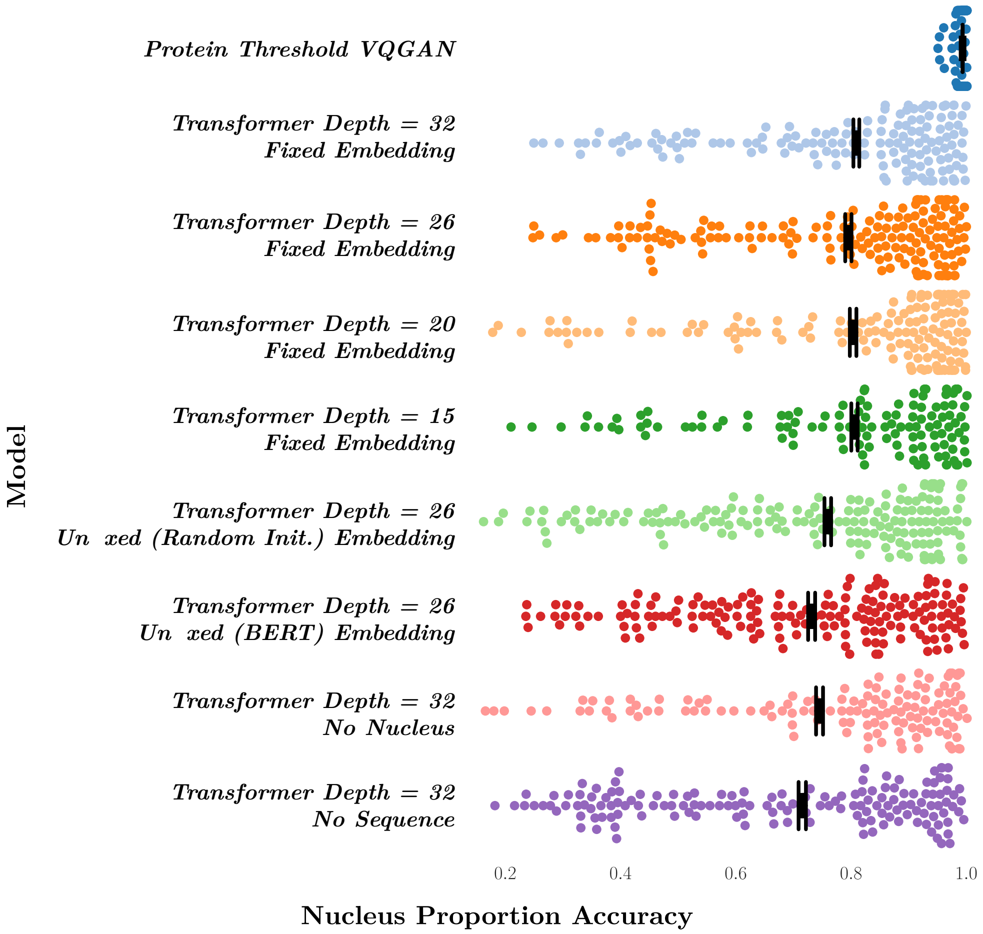

Fig. S5 Ablation Plot. "Fixed" and "Unfixed" embedding refer exclusively to the amino acid embeddings. Image embeddings are always unfixed. Mean values and standard deviation are marked in black. ~ 150 points are randomly selected for display out of 1303 total predictions per model.

descriptors come from Osorio et. al. [60], which contains amino numerical descriptions of amino acid properties from various literature sources [61–70]. Using one-hot and amino acid descriptors allowed us to scale to deeper model depths, but we did not see much improvement from doing so using these embeddings. These encodings likely do not contain sufficient information complexity about local environments that TAPE, UniRep, and ESM1b contain. While we do not see a consistent top performer on the training data, TAPE-based models generally performed the best across all metrics on the validation set, indicating a higher degree of generalizability.

Appendix S.3 Supplementary Methods

S.3.1 Dataset

Each protein entry in OpenCell is accompanied by multiple high-resolution 3D confocal images containing multiple cells [13]. Having multiple live cells enables the potential for protein distribution to be captured at several time points within a cell's lifetime. To reduce computational cost for out demonstration, we converted a 3D z-stack into a 2D maximum intensity projection [71], which still clearly depicts most subcellular structures and allow subtle subcellular protein localization differences to be distinguished from the OpenCell images [13].

The OpenCell dataset was selected because the split-fluorescent protein fusion system allows for tagging endogenous genomic proteins, maintaining local genomic context, and the preservation of native expression regulation [13]. This last point is specifically important when compared to the previously mentioned HPA, which contains $\sim 10\times$ more proteins and images. ICC-IF, which is the technique used for obtaining HPA images, requires several rounds of fixation and washing [72]. This means the proteins are not observed in a live cell, are subject to signal loss, artifacts, and/or relocalization events, and therefore does not represent the true nature of protein expression and distribution within a cell [73].

Training and validation sets were generated by randomly splitting the OpenCell dataset by protein 80%-20% training-validation For every stage of training, models were blind to sequences, nuclei, and protein images contained within the validation set. We utilize data augmentation techniques such as random horizontal and vertical flips on images during training.

S.3.2 Train-Validation Split Sequence Diversity

In machine learning applications which utilize amino acid sequence, it is recommended to cluster proteins based on similarity in order to create a distributional shift between a training and validation (and/or test) set. Oftentimes, redundancy in subsequences between both sets may results in memorization of training sequences and inflated performance metrics [74].

To investigate the effect of this on CELL-E, we performed a clustered split using a procedure identical to the one used by [8] to create, a standard dataset used in benchmarking protein localization prediction. This model relies on PSI-CD-HIT [75]. In short, we clustered proteins based on a value cutoff of a designated percentage of identity for which the alignment must cover 80% of shorter sequences. We retrained CELL-E with train/validation splits with clustered with varied threshold percentages of sequence identity, ranging from 15% to 95% for 130 epochs. Our random split used for the main CELL-E effectively represents clustering based on 100% identity.

We did not observe any patterns in cross-entropy loss during training of the main transformer model in response to different cutoff values for sequence identity.

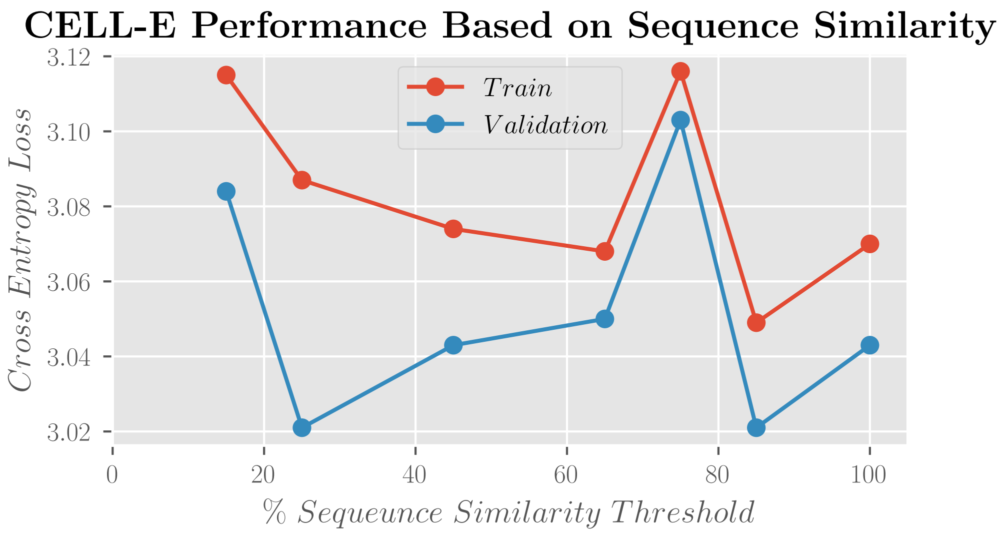

Fig. S6

S.3.3 Training

We utilized 4×NVIDIA RTX 3090 TURBO 24G GPUs for this study. 2 GPUs were utilized for training VQGANS via distributed training. Only a single GPU is ever used to train CELL-E models.

Our computer also contained 2×Intel Xeon Silver and 8×32768 mb 2933MHz DR×4 Registered ECC DDR4 RAM.

S.3.4 Nucleus Image Encoder

VQGAN code was obtained from Esser et. al. [16], which was available via MIT license (Copyright (c) 2020 Patrick Esser and Robin Rombach and Björn Ommer).

The model was trained on random 256 × 256 crops of 512 × 512 nuclei images. Adam Optimizer was used with learning rate set to 4.5×10^{-6}. The model was initially trained solely using mean-squared error reconstruction loss. After 50,000 steps, ~ 7 epochs, the discriminator loss term was introduced. This terms helps with reducing the blurriness typically associated with VAEs. 512 discrete image codes were learned. Training occurred until the model reached convergence (at 344 epochs).

S.3.5 Protein Threshold Image Encoder

The model was trained on random 256 × 256 crops of 512 × 512 nuclei images. Adam Optimizer was used with learning rate set to 4.5×10^{-6}. The model was initially trained solely using mean-squared error reconstruction loss. After 50,000 steps, ~ 7 epochs, the discriminator loss term was introduced. This terms helps with reducing the blurriness typically associated with VAEs. 512 discrete image codes were learned. Training occurred until the model reached convergence (at 371 epochs).

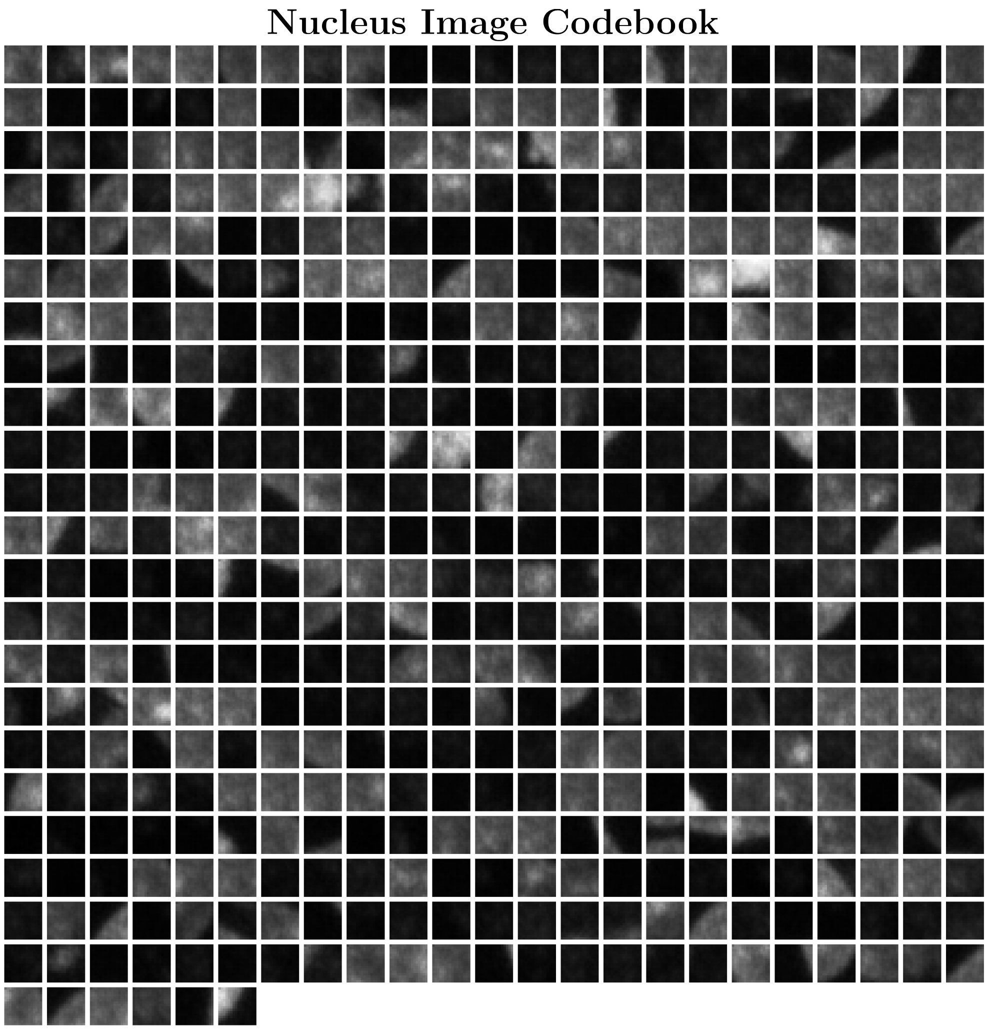

Fig. S7 512 image patches extracted from the nucleus reference VQGAN

S.3.6 CELL-E Transformer

Amino acid sequences were converted to indices via the selected language tokenizers. Unless otherwise stated, all results in this work utilized the IUPAC tokens and TAPE language embeddings. CELL-E uses encodings from TAPE, There are 30 possible codebook values for amino acids within this model, with 25 corresponding to amino acids and 5 corresponding to special tokens (i.e. padding). amino acid sequence length was limited to 1000 amino acids, which is longer than 96% of sequences within the dataset. For amino acid sequences shorter than 1000 amino acids, an end token (if utilized by the language model) was appended, followed by padding tokens. For amino acid sequences longer than 1000 amino acids, we randomly cropped a 1000 length

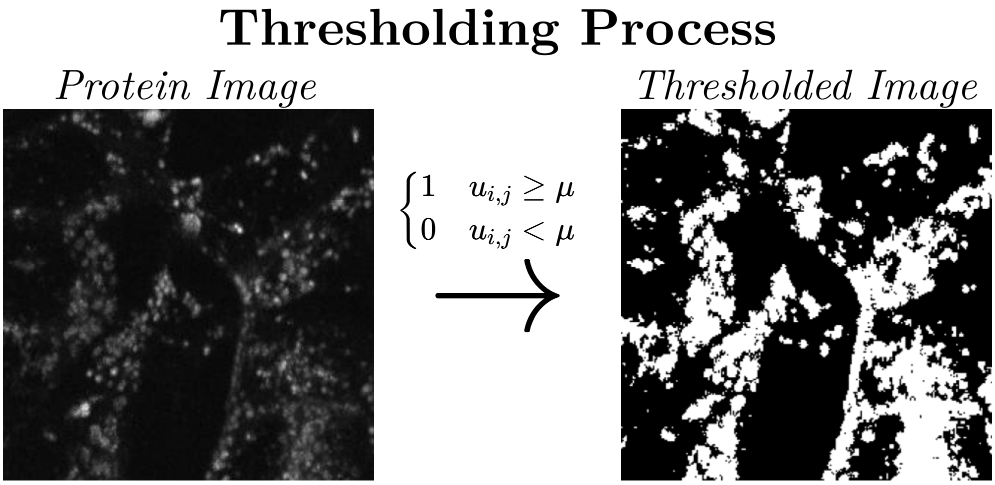

Fig. S8 Example of thresholding process to convert protein image (left) to thresholded ground truth image (right) for CELL-E model

subsection. If the right end of the crop ended before the true end of the amino acid sequence, no end token was applied. A start token is then prepended to all 1000 length sequence. The TAPE model used represents input embeddings as vectors with dimension $n\times$ 768, where n is the number of amino acids. The sequence embedding for the TAPE based models therefore had embedding vector sizes of 1001×768. Input amino acids were tokenized and their embeddings were retrieved from the language models. This input embedding is fixed. We also explored other embeddings (UniRep, ESM1b, One-hot encoding, and chemical descriptors) in Table S1.

CELL-E was trained with an Adam optimizer with learning rate set to 3×10^{-4}.

The images were passed through the encoders of their respective VQGANs to obtain codebook tokens, and the final protein threshold image token is removed. We utilized data augmentation techniques including random cropping and random flips, just as was performed when training the VQGAN models. Within the CELL-E transformer, image token embeddings were cast into the same dimensionality as the language model embedding to in order to maintain the larger protein context information, however the embeddings corresponding to the image tokens within this dimension are learned. This ultimately creates a full sequence embeddings 1512×768 (1001×768 for text, 256×768 for nucleus images and 255×768 for protein threshold images) (Fig. 2).

A rotary positional embedding [76] is then applied to the input embeddings.

We noted improved performance by shifting embeddings over by 1 (time-shifting [77]) in the feature dimension, but only for image tokens. Image token embeddings were shifted one position from the top and one position from the left.

A full attention scheme [78] is used where future tokens are masked in order to retain full sequence and image context. The output of the attention layers is passed through a block consisting of a linear and softmax layer to produce logits for predicted

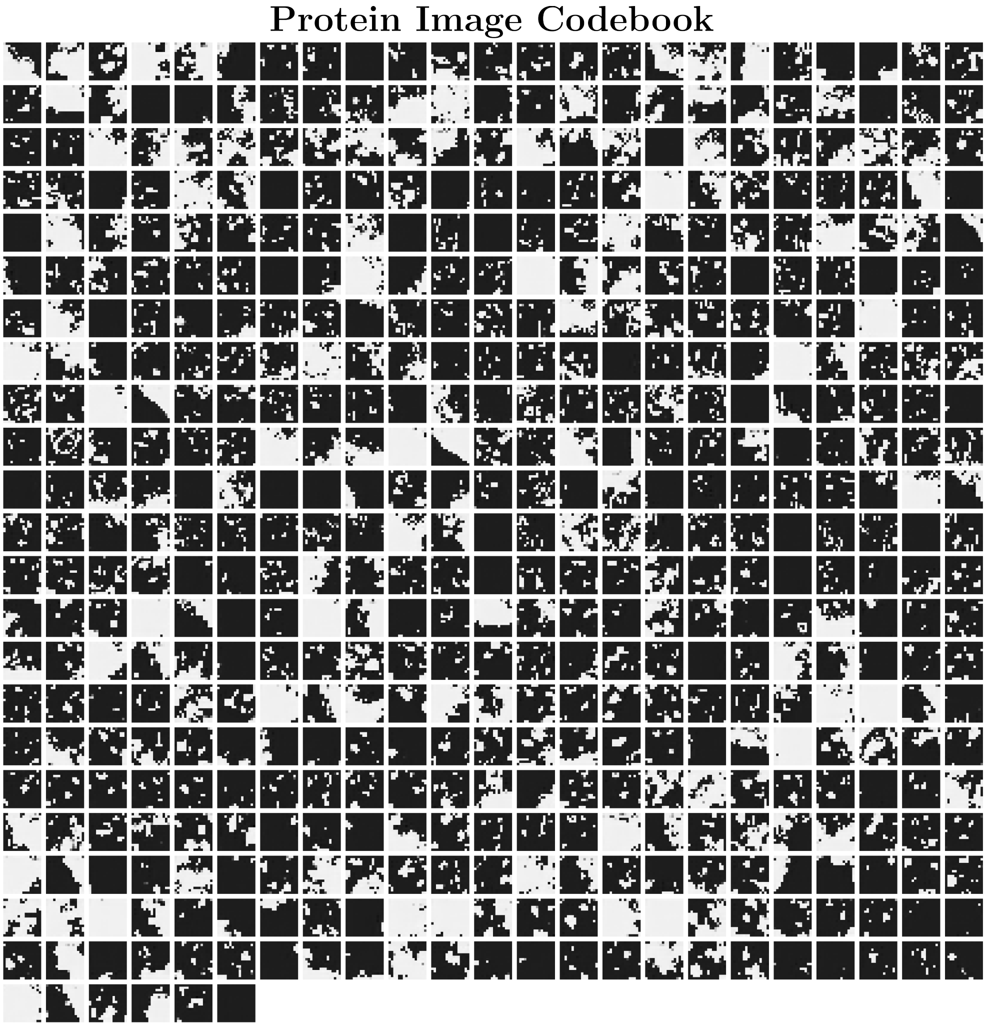

Fig. S9 512 image patches extracted from the protein threshold VQGAN

tokens at each position. Selective masking is applied so the model is unable to select anything but amino acid tokens for amino acid positions, nucleus image tokens for the nucleus image positions, and protein image tokens for the protein image positions.

Cross-entropy loss is used to measure the model's ability to reconstruct the original input vector (without the prepended start token and including the removed final protein threshold image token). The cross entropy is initially scaled by the length of the input, but further weighting is placed to emphasize the output protein image threshold tokens. We used weightings of $\frac{1}{9}$ for the amino acid tokens, $\frac{1}{9}$ for nucleus image tokens, and $\frac{8}{9}$ for the protein threshold image tokens.

Table S2 Image Accuracy

	Train		Validation	
	CELL-E	VQGAN	CELL-E	VQGAN
Nucleus Proportion Accuracy	0.94 ± 0.05	0.99 ± 0.01	0.81 ± 0.18	0.99 ± 0.01
Predicted Threshold Pixel Accuracy	0.77 ± 0.06	0.87 ± 0.08	0.77 ± 0.05	0.88 ± 0.08
Predicted 2D PDF Pixel Accuracy	0.68 ± 0.10		0.63 ± 0.10	
Structual Similarity Index Measure	0.32 ± 0.21	0.55 ± 0.25	0.25 ± 0.16	0.56 ± 0.25
Inception Score	2.77 ± 0.07	4.17 ± 0.17	2.13 ± 0.07	3.87 ± 0.17
Fréchet Inception Distance	107	15	156	23

Performance is reported as mean $\pm$ standard deviation where applicable. The VQGAN columns indicate the value of these metrics evaluated on the ground truth threshold image passed through the protein threshold VQGAN. As CELL-E selects tokens from this VQGAN to produce its outputs, these values represent the best possible performance for our model.

The main CELL-E model had a depth of 32, indicating 32 consecutive attention and feed forward blocks, and 16 attention heads with dimension = 64. We used attention and feed forward attention dropout both = .1 during training. The language embedding was fixed. Model convergence occurred at 130 epochs and these weights were used for study.

S.3.7 Performance Evaluation

To assess performance, we generated a single prediction per image found in the OpenCell set. Each image was randomly cropped and flipped similar to training, but cropped regions and flips were maintained between models.

Nucleus Proportion Accuracy

To calculate the proportion of intensity in the nucleus, we first create a mask (Fig. S10 of the nucleus channel using Cellpose [79]. We take a sum over the predicted 2D PDF pixels found within the nucleus mask, and divide this by the sum of pixels across the image.

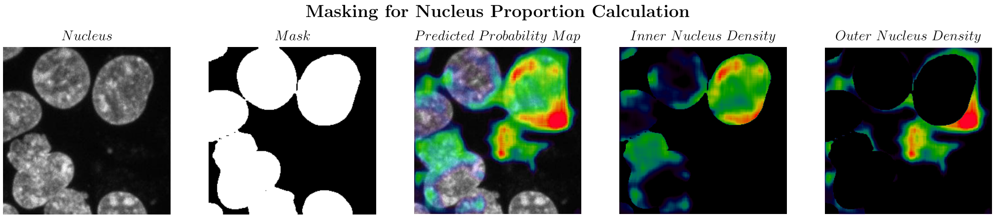

Fig. S10 Masking procedure depicted.

For the the ground truth, we use a similar masking calculation, but consider the values of the ground truth protein image. These values are subtracted to calculate

a mean-average error (MAE). Since the maximum possible value is 1 and minimum possible value is 0, we report accuracy as 1 - MAE.

Predicted Threshold Pixel Accuracy

We simple calculate a pixel-wise MAE between the predicted protein threshold image of CELL-E and the ground truth protein threshold image.

Predicted 2D PDF Pixel Accuracy

This metric is similar to Predicted Threshold Pixel Accuracy, except we evaluate the difference using the predicted 2D PDF, rather than the predicted protein threshold image. We expect this number to be less accurate as tokens with less confidence will reduce the pixel value, while all values in the protein threshold image are 0 or 1.

SSIM

Structural similarity index measure (SSIM) is a measure of local perceptual similarity between images. It considers neighboring pixels to evaluate loss contextually by incorporating luminance and contrast information. SSIM values range between 0, indicating no similarity, and 1, indicating maximum similarity.

IS

Inception score (IS) is often used to evaluate the image outputs of GANs as a measure of "realisticness." It rewards image variety and similarity to real-life data. Performance evaluation is based on the magnitude of the IS score.

FID

Fréchet Inception Distance (FID) is another popular metric for evaluating the quality of images from generative models. It compares the distributions between generated and ground truth images as the squared Wasserstein metric between two multidimensional Gaussian distributions. For this study FID was scored against the training or validation sets when applicable, rather than the entire OpenCell dataset.

Nuclear Localization Prediction

The ground truth label for nuclear localization was designated by masking the nucleus, but computing the proportion of intensity on the ground truth thresholded protein image. If $> 50\%$ of this intensity was contained within the area of the nuclear mask, the assigned label would be positive for nuclear localization. Otherwise, the protein would be designated as non-nuclear. For the predicted label, we took a summation over the masked and unmasked regions of the predicted 2D PDF. If $> 50\%$ of pixel intensity for the 2D PDF was found in the nucleus, it was classified as a nuclear localizing protein. The protein localization prediction models were provided the amino acid sequence and were considered to predict nuclear if present in the localization prediction. These predictions were also compared against our naïve labels.

S.3.8 Visualizing Attention

To obtain Fig. 6, we first split the 16×16 generated threshold image patches into 2 groups, one where protein is primarily determined to be present $\bar{u}_{i,j} > .75$ and another where background tokens are primarily selected $\bar{u}_{i,j} < .25$. For each respective group, we calculate a median of the attention matrices and used attention rollout [80] to recursively multiply across 32 layers. The final layers of both groups are then compared. We initially look at tokens with higher weightings for the present protein group, and discard the rest.

We show the entire image generation process, with frames corresponding to time-steps, in the attached video file: DNAtopoisomerase1.mp4.

References

[1] Hu, T., Chitnis, N., Monos, D. & Dinh, A. Next-generation sequencing technologies: An overview. *Human Immunology* **82**, 801–811 (2021). URL https://www.sciencedirect.com/science/article/pii/S0198885921000628.

[2] Palma, C.-A., Cecchini, M. & Samorì, P. Predicting self-assembly: from empirism to determinism. *Chemical Society Reviews* **41**, 3713–3730 (2012). URL https://pubs.rsc.org/en/content/articlelanding/2012/cs/c2cs15302e. Publisher: The Royal Society of Chemistry.

[3] Chacinska, A., Koehler, C. M., Milenkovic, D., Lithgow, T. & Pfanner, N. Importing Mitochondrial Proteins: Machineries and Mechanisms. *Cell* **138**, 628–644 (2009). URL https://www.sciencedirect.com/science/article/pii/S0092867409009672.

[4] Imai, K. & Nakai, K. Prediction of subcellular locations of proteins: where to proceed? *Proteomics* **10**, 3970–3983 (2010).

[5] Ahmed, H. R. & Glasgow, J. Sokolova, M. & van Beek, P. (eds) *A Novel Particle Swarm-Based Approach for 3D Motif Matching and Protein Structure Classification.* (eds Sokolova, M. & van Beek, P.) *Advances in Artificial Intelligence*, Lecture Notes in Computer Science, 1–12 (Springer International Publishing, Cham, 2014).

[6] Gardy, J. L. & Brinkman, F. S. L. Methods for predicting bacterial protein subcellular localization. *Nature Reviews Microbiology* **4**, 741–751 (2006). URL https://www.nature.com/articles/nrmicro1494. Bandiera_abtest: a Cg_type: Nature Research Journals Number: 10 Primary_atype: Reviews Publisher: Nature Publishing Group.

[7] Lu, J. *et al.* Types of nuclear localization signals and mechanisms of protein import into the nucleus. *Cell Communication and Signaling* **19**, 60 (2021). URL https://doi.org/10.1186/s12964-021-00741-y.
